# A Molecular Genetic Basis Explaining Altered Bacterial Behavior in Space

**DOI:** 10.1371/journal.pone.0164359

**Published:** 2016-11-02

**Authors:** Luis Zea, Nripesh Prasad, Shawn E. Levy, Louis Stodieck, Angela Jones, Shristi Shrestha, David Klaus

**Affiliations:** 1 BioServe Space Technologies, Aerospace Engineering Sciences Dept., University of Colorado, Boulder, CO, United States of America; 2 Genomic Services Laboratory, HudsonAlpha Institute for Biotechnology, Huntsville, AL, United States of America; 3 Department of Biological Science, University of Alabama in Huntsville, Huntsville, AL, United States of America; National Renewable Energy Laboratory, UNITED STATES

## Abstract

Bacteria behave differently in space, as indicated by reports of reduced lag phase, higher final cell counts, enhanced biofilm formation, increased virulence, and reduced susceptibility to antibiotics. These phenomena are theorized, at least in part, to result from reduced mass transport in the local extracellular environment, where movement of molecules consumed and excreted by the cell is limited to diffusion in the absence of gravity-dependent convection. However, to date neither empirical nor computational approaches have been able to provide sufficient evidence to confirm this explanation. Molecular genetic analysis findings, conducted as part of a recent spaceflight investigation, support the proposed model. This investigation indicated an overexpression of genes associated with starvation, the search for alternative energy sources, increased metabolism, enhanced acetate production, and other systematic responses to acidity—all of which can be associated with reduced extracellular mass transport.

## Introduction

Since the Soviet Korabl-Sputnik 2 (called Sputnik 5 in the West) and the American Discover 17 satellites launched in 1960, numerous bacterial experiments have taken place in Earth’s orbit. It is now known that bacteria grown in space exhibit a number of differences relative to their Earth behavior. For non-motile, suspension cultures in particular, general trends of reduced lag phase and increased final population density have been consistently observed [1]. Other experiments have indicated changes such as improved biofilm formation [2,3], higher specific productivity of secondary metabolites [4], a thicker cell envelope [5] and enhanced conjugation efficiency [6]. In addition to these various altered microbial growth characteristics, results indicating increased capability to cause disease (virulence) [7,8] and reduced susceptibility to antibiotics in space have also been reported [9–15]. These health-related findings present especially concerning challenges for long duration space crews in terms of treating potential infections.

Although the cause of each of these observations is commonly attributed to some aspect of the reduced-gravity environment, a validated model quantifying the specific underlying mechanisms has not yet been established. Early theoretical analyses suggested that intracellular processes are not likely to be directly affected by gravity at this scale [16]. It has been hypothesized that the extracellular environment is altered due to the lack of gravity-driven forces and flows, namely buoyancy, sedimentation, and convection. Thus, molecular transport through the boundary layer to and from the cell essentially becomes limited to diffusive processes only [17–22]. This subtle, quasi-stable change in the chemical environment in the immediate vicinity of the cell (as an osmotic solute gradient) has been hypothesized to subsequently give rise, at least in part, to most of the altered behaviors summarized above [9,10,23–26]. Ground simulations of microgravity via clinorotation and the engineering of mutant buoyant strains have resulted in similar trends as observed in space experiments [27–30]. Interestingly, bacterial motility has been shown to eradicate many of these altered responses, presumably by disrupting the otherwise quiescent fluid environment experienced in the boundary layer surrounding non-motile cells in space [31].

However, to date neither spaceflight results, ground-based studies, physical measurement techniques [32] nor computational approaches [22], have provided sufficient evidence needed to definitively confirm the extracellular transport model proposed to govern bacterial responses to spaceflight. Therefore, although the above results and associated explanations all suggest that reduced mass transport can be responsible for the reported changes, they do not yet confirm this theory. In order to gain more insight into this suspected transport-driven underlying cause, we designed an experiment to characterize gene expression differences between matched *E*. *coli* liquid suspension cultures grown in space and on Earth. We hypothesized that for *E*. *coli* cultures grown in space, the genes associated with starvation (due to reduced glucose uptake) and acidic response (from buildup in the local microenvironment) would be overexpressed with respect to matched Earth controls. These responses would be expected to occur with reduced extracellular mass transport into and away from the cell. Because transcriptomic analyses allow us to investigate the dynamic responses of cells at the molecular level, these gene expression data could provide insight into environmental factors that the bacterial cells were exposed to in space and corroborate the proposed altered extracellular environment model.

## Results

**Fig 1:**
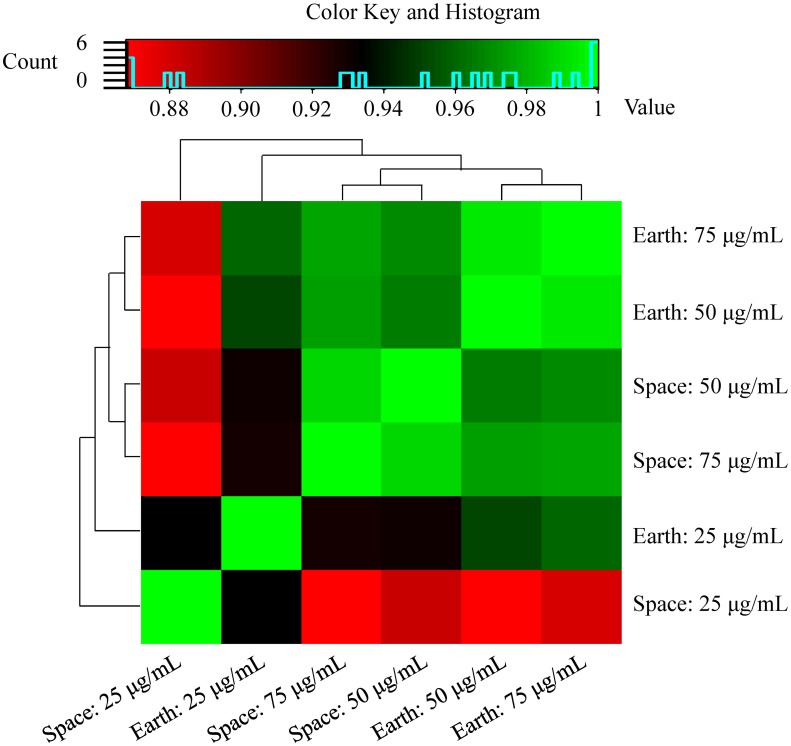
Spearman correlation. The hierarchical clustering of Spearman correlation coefficients showing pairwise comparisons of effect of Gentamicin Sulfate exposure at various concentrations to *E*. *coli* grown in space and on Earth. This observation indicates that transcriptome-wide RNA expression profiles may be used to distinguish and characterize the response of *E*.*coli* to antibiotic concentration and gravitational conditions at molecular levels.

Three groups of samples were used for this analysis: *E*. *coli* challenged with 25, 50 or 75 μg/ml of Gentamicin Sulfate, each group having four replicates cultured in space and four on Earth (with the exception of Space and Earth 50 μg/ml, and Space 75 μg/ml, which had *n = 3*). A Welch’s test showed that there was a 13-fold increase in overall final cell count in space with respect to ground (*Welch’s F*(1, 10.88) = 116.68, *p* <0.0001). The no-drug controls were fixed at a different time than the drug samples so the “drug concentration” independent variable can’t be properly assessed. Nonetheless, the other independent variable, gravity– μg, having Earth 1g as control—can be interrogated. Furthermore, analysis based on pairwise comparison of averaged RNA expression among these groups by the Spearman correlation coefficient showed well-defined segregation in the collective mRNA expression levels between *E*.*coli* grown in Space and Earth (Fig 1).

The number of genes that were differentially expressed per group is described in S1 Table. Gene expression analyses showed that 81 genes were commonly overexpressed throughout the three tested groups (see S2 Table) (a differential expression larger than |2| was consider statistically significant). Table 1 presents the genes from this list that were overexpressed at least a 10-fold. It was noted that there were no trends in the differential expression of most genes with respect to drug concentration.

**Table 1:** Overexpressed genes in all three sets. Commonly overexpressed genes throughout the three test groups (25, 50 and 75 μg/ml Gentamicin Sulfate), where genes were up-regulated at least ten-fold in space with respect to their matched Earth (1g) controls in one or more of the tested conditions. S11 Table provides a Gene set analysis of these genes in pathways and with statistical significance.

Gene Name	Known or predicted function ^a^	Molecular Function ^a^	Biological Process ^a^	25 μg/mL^b^	50 μg/mL^b^	75 μg/mL^b^
*entS_2*	Enterobactin exporter EntS*		enterobactin transport	2.36	14.44	2.45
*gadX*	HTH-type transcriptional regulator GadX	Catalytic activity / Nucleic acid binding	Metabolism / Biological regulation / Response to stimulus	13.73	5.82	2.05
*hdeA*	Acid stress chaperone HdeA			6.24	28.09	2.66
*hdeB*	Acid stress chaperone HdeB			6.10	29.08	2.68
*hdeD*	Protein HdeD			3.04	14.86	2.98
*thiE*	Thiamine-phosphate synthase	Catalytic activity	Metabolism / Cellular Process	2.40	28.59	6.57
*thiF*	Sulfur carrier protein ThiS adenylyltransferase	Catalytic activity	Metabolism / Cellular Process	2.41	28.87	7.44
*thiG*	Thiazole synthase			2.26	30.48	8.04
*thiH*	2-iminoacetate synthase	Catalytic activity	Metabolism	2.20	24.88	7.44
*thiS*	Sulfur carrier protein ThiS			2.49	32.41	6.01
*yccJ*	Uncharacterized protein YccJ			28.70	7.22	2.13
*yegP*	UPF0339 protein YegP			23.93	5.91	2.21
*yhiD*	Uncharacterized protein YhiD			6.67	19.57	2.16
*yiaG*	Uncharacterized HTH-type transcriptional regulator YiaG			14.22	8.45	4.36
*yjdI*	Uncharacterized protein YjdI			6.26	12.76	3.06

^a^ Based on the PANTHER (Protein ANalysis Through Evolutionary Relationships) Classification System database as of August 6, 2015 ref [48].

^b^ The fold-increase was acquired as the ratio of the average of the spaceflight samples expressions over the average of the Earth (1g) control samples expressions.

* This is the known or predicted function for the collective *entS* gene and not only for the *entS_2* isoform.

Table 1 shows that two families of genes constitute over half of the commonly-up-regulated genes that were expressed at least 10-fold: *thiEFGHS* and *hdeABD*. The *thi* genes were overexpressed in between 2.20x and 32.41x in the spaceflight samples. *thiEFGH* are the structural genes for Thiamine biosynthetic enzymes while *thiS* is a sulfur donor in that process [33]. The overexpression of these genes indicates that there was an increased synthesis of thiamine, which is needed for carbohydrate metabolism [34]. The enhanced production of thiamine suggest that the cells were under starvation conditions, as *E*. *coli* accumulates adenosine thiamine triphosphate (AThTP), a form of thiamine, in response to lack of energy substrates [35]. The *hde* family of genes (overexpressed in between 2.66x and 29.08x) is associated with acidic conditions. For example, *hdeA* synthesizes a protein (HdeA) that combats the deleterious effects of acid on periplasmic proteins, *hdeB* is associated with cell envelope modifications [36], and *hdeD* is known to be acid-induced [37]. This differential gene expression insight suggests two primary underlying factors that support how the altered extracellular environment model is hypothesized to govern bacterial behavior in space: starvation and acidic conditions.

**Fig 2:**
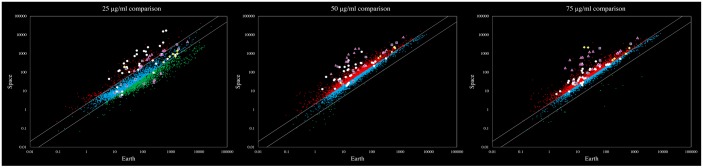
Differential Gene Expression. Scatter plots showing averaged gene expression (log_10_ scale) in *E*.*coli* as detected by RNA-seq between space (*y*-axis) and Earth (*x*-axis) at various Gentamicin Sulfate concentrations: 25 μg/mL (A); 50 μg/mL plot (B); and 75 μg/mL (C). Red dots indicate overexpression, blue non-differential expression, and green underexpression of genes in space with respect to Earth (1g) controls. Pink triangles show genes associated with metabolism but not directly involved in the glucose catabolism pathways (listed in S6 Table); white circles indicate the expression of genes associated with acid resistance; yellow diamonds indicate the genes associated with acetate production from glucose (listed in S7 Table); and purple squares represent the genes induced by acetate but not by acidity (listed in S8 Table).

Fig 2 shows gene expression in space with respect to Earth, for each of the tested groups, where each dot represents a gene (totaling 4,313 analyzed genes). The figure shows the (i) overexpression of genes associated with metabolism of substrates that were not present and the (ii) overexpression of genes associated with conferring *E*. *coli* with resistance to acidity– indicative of starvation conditions and a reduction in pH in the extracellular environment, respectively.

### Observed Starvation and Stimulated Metabolism

Gene expression data suggest that cells in space were under starvation conditions, as several response mechanisms *E*. *coli* cells implement under these circumstances were observed in the spaceflight samples. When substrate concentration decreases, cells devote more of their limited resources to the genome transcription needed to broaden the search for alternative sources of carbon, even if these are unavailable. This is sometimes paralleled to the “risk-prone foraging” strategy used by some starving animals, where they tend towards high-risk behaviors in their search for food. It is believed bacteria do this to increase their ability to rapidly switch carbon catabolic pathways if a new substrate becomes available [38].

The systematic overexpression of the *thiFGHS* genes indicate that the spaceflight cells were experiencing a lack of available substrate compared to their matched ground controls [35]. This indication is supported by the activation of the *malE* gene in space in a 43.80-fold and 36.07-fold in the 50 μg/mL and 75 μg/mL sets, respectively (this was the single most overexpressed gene in the 75 μg/mL set, as seen in S5 Table). *malE* codes the MalE protein, which is essential for the transport of maltose through the inner cell membrane [39]. The encoding of the MalE protein is under a positive control, meaning that its synthesis is inducible by maltose [40]. However, in this experiment the source of carbon was Glucose (*C*_6_*H*_12_*O*_6_), while maltose—a more complex molecule (*C*_12_*H*_22_*O*_11_)–was never introduced into the growth medium. This observation is complemented by the overexpression of *lamB*, which synthesizes LamB—a protein that facilitates the diffusion of maltose across the outer membrane [41]. *lamB* was overexpressed 23.63x and 30.05x in the 50 and 75 groups, respectively. The overexpression of *malE* and *lamB* despite the lack of maltose suggests that, in space, cells were broadening the search for alternative sources of carbon even if they were unavailable, similar to the “risk-prone foraging” principle described in ref. [38], and characteristic of carbon-starved cultures.

Investigating the overexpressed genes pointed at an increase in activity related with trans-membrane influx of substrates. After *malE*, the most obvious of these cases was *malK*, a gene that synthesizes an oligopeptide permease protein. *malK* was overexpressed in the 50 and 75 μg/ml sets to 22.39x and 31.81x, respectively (in the 75 μg/ml set, *malK* was the second most up-regulated gene, after *malE*, as seen in S5 Table). Another case is presented by the overexpression of the *oppAB* (overexpressed in the 25 and 50 μg/ml sets in between 3.45x and 5.87x) and *oppCDF* (overexpressed in all the three sets in between 2.06x and 4.77x) genes in space. The *opp* genes synthesize proteins related with oligopeptide transport—used by *E*. *coli* and *Salmonella typhimurium* as a source of carbon and energy—into the cell together with the OmpC and OmpF porins [42]–*ompC* and *ompF* were overexpressed up to 2.80x and 9.84x, respectively (in the 50 μg/ml set). This suggests that the cells in space may have been trying to increase the influx rate of any potential source of energy, again, suggesting starvation. This is further supported by the overexpression (7.23x and 3.60x in the 25 and 50 μg/ml sets, respectively) of *dps*, a starvation-induced gene that synthesizes Dps, a DNA-protection protein [43]. Furthermore, *crp*, a gene associated with glucose starvation was overexpressed in space (3.49x and 2.23x in the 50 and 75 μg/ml sets, respectively), as were *glnG* (2.91x and 2.05x in the 50 and 75 μg/ml sets, respectively) and *nac* (2.70x in the 50 μg/ml set), the latter two associated with nitrogen-starvation [44].

**Fig 3:**
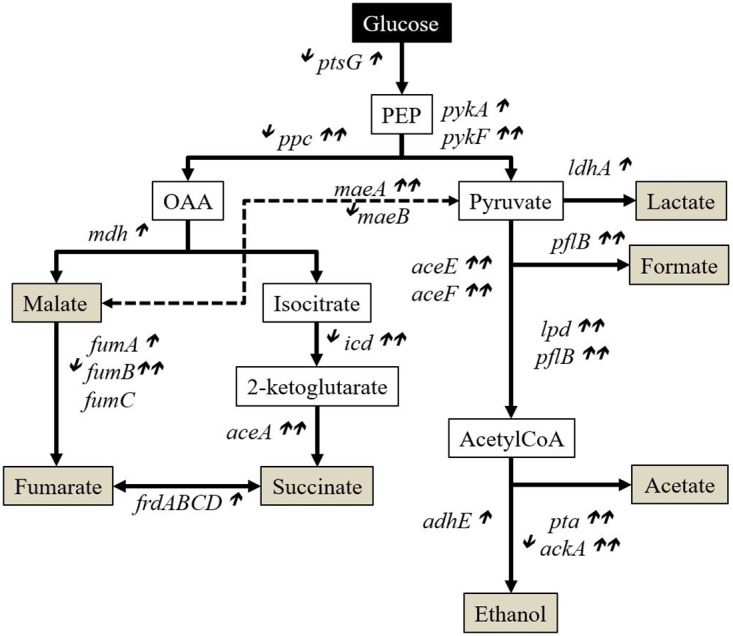
Genes involved in the TCA and Glyoxylate Cycles. *E*. *coli* metabolic pathways, compiled from ref. [45] and [46]. Most of the genes associated with these metabolic pathways were overexpressed in the spaceflight samples with respect to their matched Earth (1g) controls. ^⬇^Underexpressed in one of the three groups;^⬆^overexpressed in one group; ^⬆⬆^overexpressed in two groups; ^⬆⬆⬆^overexpressed in all three groups.

The trend of the overexpressed genes to be associated with starvation and enhanced trans-membrane influx activity in space led us to wonder if there were also changes in the activation of genes related with glucose catabolism, since this molecule was the carbon source in these cultures. Fig 3 describes *E*. *coli*’s central Glucose metabolism pathways (tricarboxylic acid (TCA) and Glyoxylate Cycles) and their respective genes, based off ref. [45] and [46]. From these 27 genes, we had data for 26 (we did not find the expression of *frdB* (b4153) in the *E*. *coli* (DH10B) sequence that was downloaded from the UCSC genome browser) and, from these 26, we observed that 18 were overexpressed in space (69%), 5 showed mixed results, while only 3 were non-differentially expressed (see Table 2).

**Table 2:** Genes involved in the TCA and Glyoxylate Cycles. Differential expression in space with respect to matched Earth (1g) controls for the three tested scenarios. S12 Table provides a Gene set analysis of these genes in pathways and with statistical significance.

Gene Name	Reaction Catalyzed by the Gene’s product ^a^	25 μg/mL	50 μg/mL	75 μg/mL	25	50	75
*aceA*	2-ketoglutarate →Succinate	-1.64	2.97	2.16			
	Isocitrate → Glyoxylate						
*aceB*	Glyoxylate → Malate	-1.27	4.03	2.85			
*aceE*	Acetate production (Pyruvate → AcetylCoA)	-1.69	3.42	2.35			
*aceF*	Acetate production (Pyruvate → AcetylCoA)	-1.27	3.57	2.52			
*ackA*	AcetylCoA → Acetate	-3.65	3.72	2.17			
*adhE*	AcetylCoA → Ethanol	1.85	5.73	3.47			
*frdA*	Fumarate ↔ Succinate	1.04	2.30	1.40			
*frdB*	Fumarate ↔ Succinate	#N/A	#N/A	#N/A			
*frdC*	Fumarate ↔ Succinate	1.57	2.42	1.44			
*frdD*	Fumarate ↔ Succinate	1.52	2.04	1.13			
*fumA*	Malate → Fumarate	1.08	2.97	1.98			
*fumB*	Malate → Fumarate	-2.79	2.85	2.75			
*fumC*	Malate → Fumarate	-1.35	1.47	1.48			
*icd*	Isocitrate → 2-ketoglutarate	-2.32	3.95	2.99			
*ldhA*	Pyruvate → Lactate	-1.57	2.15	1.23			
*lpd*	Acetate production (Pyruvate → AcetylCoA)	-1.20	2.91	2.20			
*maeA*	Malate ↔ Pyruvate	-1.20	3.50	2.02			
*maeB*	Malate ↔ Pyruvate	-2.70	1.52	-1.04			
*mdh*	OAA → Malate	-1.48	3.05	1.75			
*pflB*	Acetate production (Pyruvate → AcetylCoA)Pyruvate → Formate	-1.06	5.07	4.04			
*ppc*	PEP → OAA	-3.05	6.53	6.18			
*poxB*	Acetate production (Pyruvate → Acetate)	8.77	3.48	1.79			
*pta*	AcetylCoA → Acetate	-1.56	4.31	2.97			
*ptsG*	Glucose → Glucose-6-phosphate	-2.06	2.41	1.49			
*pykA*	PEP → Pyruvate	-1.32	3.38	1.83			
*pykF*	PEP → Pyruvate	-1.62	3.61	2.02			

^a^ Mostly from ref. [45].

The last three columns are graphical indicators of non-differential expression (white cells), over- (black cells), and under-expression (light gray cells). A differential expression larger than |2| was consider statistically significant.

A list of genes associated with metabolism but not directly involved in the glucose catabolism pathways discussed in Fig 3 was compiled from ref. [45] and [46]. From the list of 23 genes, 17 were overexpressed in space (74%) (see S6 Table). All of these data indicate that overall metabolic activity was stimulated in space—which suggests that this may be the mechanism responsible for the typically observed increase in bacterial cell concentrations in microgravity.

### Observed Enhanced Acid Production

Gene expression data also indicated that there was an increased production of organic acids in space. Several genes associated with acid formation, beyond the ones discussed before in this study, were overexpressed, including the *trpABCDE* operon. The biological process associated with these genes is cellular amino acid biosynthesis through tryptophan and the subsequent formation of amino acids and organic acids [47,48]. Although these genes were underexpressed in the 25 μg/mL group (from -2.06x to -3.65x), they were overexpressed in between 45.61x and 69.11x in the 50 μg/mL set and in between 18.74x and 30.14x in the 75 μg/mL group. In the 50 μg/mL group, *trpABCDE* were the top-5 most up-regulated genes (see S4 Table) while in the 75 μg/mL set they were the highest overexpressed family of genes after *malE* and *malK* (see S5 Table).

Other genes associated with glucose catalysis into acetate were also activated in the spaceflight cultures. As a matter of fact, all of the genes known to us to synthesize the enzymes responsible for converting pyruvate to acetyl compounds were overexpressed in space: according to ref.[46] under anaerobic conditions this process is controlled by pyruvate formate lyase Pfl (*pflB*), and pyruvate oxidase PoxB (*poxB*), where the encoding gene is in parenthesis. Ref. [44] also shows that glucose can be metabolized directly into acetate through PoxB, by catalyzing the decarboxylation of pyruvate to acetate and CO_2_. Based on ref. [45] and [46], a list of 8 genes associated with acetate production from Glucose was compiled (see S7 Table). From this group, 7 genes were overexpressed in space (88%) in between 2.02x and 8.77x. It is noteworthy that, although ref. [44] links *poxB* overexpression with phosphate starvation, *phoB*, a gene that is up-regulated by lack of phosphate sources, was not overexpressed in the spaceflight samples and was actually underexpressed (-2.98x) in the 25 μg/mL group. In other words, we observed systematic responses to carbon and nitrogen starvation but only partial to phosphate starvation.

### Observed Activated Responses to Acidic Conditions

Besides the stimulated metabolism discussed before, the analysis of the overexpressed genes in space also revealed activated responses to highly acidic conditions. *E*. *coli* has three systems for acid resistance (AR). AR1 was not applicable to the testing conditions in AES-1 as it is glucose-repressed [49]. AR3, the arginine-dependent system governed by *adiA*, *adiC*, and *adiY* [50], was not assessable because we did not find the expression of *adiA* or *adiY* in the *E*. *coli* (DH10B) sequence that was downloaded from the UCSC genome browser. However, *adiC* was overexpressed in space (3.46x and 9.50x in the 25 μg/mL and 50 μg/mL sets, respectively). The glutamate-dependent AR2, the most effective of the three acid resistance systems, was systematically activated in space. AR2 is governed by the *gadA* and *gadBC* genes [36], which in turn are regulated by *gadE* and, to a lesser degree, by *gadX* and *gadW* [49,50], all of them overexpressed in the spaceflight samples in between 2.05x and 25.60x, as seen in Table 3.

**Table 3:** Differential expression of acid resistance genes. Differential expression of the genes associated with acid resistance, and their categories as part of the acid resistance system 2 (AR2) (from ref. [49]), characterized to be partly RpoS-dependent (from ref. [51]), or up-regulated by acetate (from ref. [36]), as applicable. The last three columns are graphical indicators of non-differential expression (white cells), over- (black cells) and under-expression (light gray cells). S13 Table provides a Gene set analysis of these genes in pathways and with statistical significance.

Gene Name	AR2	Partly RpoS-dependent	Up-regulated by acetate	25 μg/mL	50 μg/mL	75 μg/mL	25	50	75
*arnB*				-2.00	9.28	5.58			
*arnC*				-1.30	8.05	2.95			
*asr*				-1.70	9.75	1.78			
*cbpA*				3.69	3.70	1.81			
*cfa*			*x*	1.87	1.79	1.65			
*dctR*				6.78	6.29	2.36			
*dps*			*x*	7.23	3.60	1.66			
*gadA*	*x*	*x*	*x*	14.07	17.49	1.18			
*gadB*	*x*	*x*	*x*	12.22	25.60	1.90			
*gadC*	*x*	*x*	*x*	14.18	11.11	1.74			
*gadE*	*x*	*x*		23.47	23.04	1.71			
*gadW*	*x*	*x*	*x*	8.33	5.69	2.25			
*gadX*	*x*	*x*	*x*	13.73	5.82	2.05			
*hdeA*		*x*	*x*	6.24	28.09	2.66			
*hdeB*		*x*	*x*	6.10	29.08	2.68			
*hdeD*		*x*	*x*	3.04	14.86	2.98			
*hpf*			*x*	-2.57	2.74	1.77			
*mdtE*		*x*		5.97	1.66	1.16			
*ompC*			*x*	-1.05	2.80	1.33			
*osmC*			*x*	-1.48	1.75	1.24			
*osmY*			*x*	1.93	3.23	1.53			
*slp*		*x*	*x*	14.78	9.52	1.78			
*wrbA*				24.76	7.66	1.76			
*yahO*				#N/A	#N/A	#N/A			
*ybaS*				8.39	2.62	1.33			
*ybaT*				4.80	1.54	1.44			
*ycaC*				6.97	5.92	2.42			
*yccJ*			*x*	28.70	7.22	2.13			
*ydiZ*				3.12	4.58	3.85			
*yeaQ*			*x*	9.95	6.26	3.80			
*yebV*				1.96	3.71	1.75			
*ygfQ*				1.25	1.49	1.67			
*yhiM*				6.91	6.94	2.10			
*yiaG*				14.22	8.45	4.36			
*yjfC*				-2.96	1.75	1.27			

The main regulator of the AR2 systems is GadE, a protein encoded by the *gadE* gene, which can be activated at acidity levels of pH 2 [49,50]. Ref. [50] provides a literature review that summarizes how AR2 allows *E*. *coli* cells to survive highly acidic conditions (pH 2). Ref. [52] demonstrated that through this mechanism, *E*. *coli* cells growing in a pH 2.5 extracellular environment were able to maintain a 4.2 ± 0.1 internal pH. This is an increase from the eventually-lethal 3.6 internal pH they would experience otherwise [44]. All six of the AR2 genes (*gadA/BC/E/W/X*) were overexpressed in spaceflight (100%). The *evgAS*/*ydeO* circuit was not taken into account in this analysis as these genes don’t play a role in AR2 at stationary phase [50].

Ref. [51] presented a list of eleven RpoS-regulated acid response genes that include *gadA/BC/E/W/X* and *hdeABD*. All of these genes (which ref. [37] categorized as an “acid-fitness island”) were systematically overexpressed on the spaceflight samples. The other two genes from this list presented by ref. [51] are *slp* and *mdtE*. *slp* was up-regulated (14.78x and 9.52x in the 25 μg/mL and 50 μg/mL sets, respectively), and *mdtE* was also overexpressed (5.97x in the 25 μg/mL group). Thus, from the 11 RpoS-regulated acid reponse genes, all 11 (100%) were overexpressed in space. This suggests that, in space, the acidity in/around the cell was increased. However, *rpoS* was not up-regulated (it was actually underexpressed in one set in a -2.27-fold), which further supports the acidity increase, as these genes’ dependence on RpoS is reduced or even abolished under acid stress conditions [51] (thus called “partly” RpoS-dependent in Table 3).

Another set of genes that was systematically overexpressed in space were those that are up-regulated by the presence of acetate. As seen in Table 3, from the 35 acid-induced genes, 17 are also induced by acetate [36]. From these 17 genes, 14 were overexpressed in space (82%) in between 2.05x and 29.08x, while only one gene was underexpressed in one set (but overexpressed in another). These results led to the question of whether the observed reaction to acidity was due in part to acetate, but a conclusion could not be definitively reached since these genes are induced by both acetate and overall acidity. To try to find an answer to this, we compared a list of 26 acetate-induced genes published in ref. [53] against the list of acidity-induced genes presented by ref. [36] and we identified 9 genes that are induced by acetate but not by overall acidity (S8 Table). Seven out of the nine genes in this group were overexpressed in space (78%) in between 2.18x and 10.50x, while one gene was both up- and down-regulated. Altogether, from the 26 acetate-induced genes presented in ref. [53], 23 were overexpressed in space (88%). All of these data sets together point to a potential cause for the decrease in pH that triggered the systematic acid-response mechanisms of *E*. *coli*: the increase in acidity may have been in part due to a rise in acetate concentration in and/or around the cell. This is in agreement with the observed increase in acetate production, indicated by the overexpression of 88% of the genes associated with acetate synthesis from Glucose (see S7 Table), and with the extracellular acid buildup hypothesized to govern several observed bacterial responses in space as noted above.

Comparing the genes identified by refs. [36], [49], and [51], a set of 16 genes were identified as being associated with acid resistance but not to either the “AR2”, “partly RpoS-dependent” or “induced by acetate” categories, and are listed on Table 3. From these 16 genes, we have data for 15 (we did not find the expression of *yahO* (b0329) in the *E*. *coli* (DH10B) sequence that was downloaded from the UCSC genome browser), 12 of which were overexpressed (80%) in the spaceflight samples in between 2.10x and 24.76x. One of these genes, *wrbA*, was the second gene overexpressed the most (24.76x, only after *yccJ*, another acid-associated gene) in the 25 μg/mL samples (S3 Table). Overall, from the 35 acid resistance-associated genes (Table 3), 28 (80%) were overexpressed in space, further indicating that, in space, the cells were living in an increased acidic environment.

### Mixed Bulk Fluid pH

The final question was whether the observed overexpression of the acid resistance genes was due to a localized acid build-up around the cell or in response to an overall increased acidity of the bulk fluid. Since it is not possible to measure the boundary level pH directly, the observed genetic acid response was intended to serve as a proxy for this local environment. By measuring the pH of the mixed bulk fluid post flight, it was concluded using two-factor ANOVAs that there was no statistically significant difference between the space and Earth samples. As would be expected under conditions of reduced extracellular transport, this therefore suggests that the observed genetic response was likely due to an increase in pH in the quasi-stable boundary layer as a result of localized, byproduct buildup in space, as predicted by the model, and not from generally increased overall acidic conditions in the mixed bulk fluid.

## Discussion

**Fig 4:**
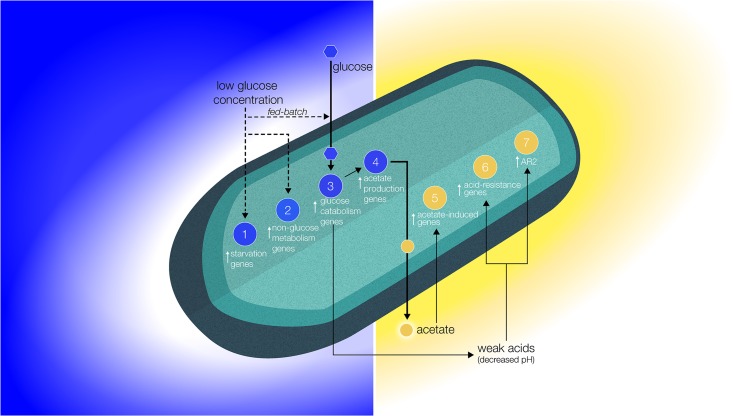
Altered Extracellular Model. Biomolecular model based on the gene expression data analyses support the reduction of glucose molecules (blue gradient) and acid buildup (gold gradient) proposed to occur in the boundary layer around the cell. This altered extracellular environment has been hypothesized to result as an effect of reduced gravity-driven forces acting on the cell-fluid system and has been put forth as the biophysical mechanism governing bacterial behavior in space. Blue circles indicate overexpression of genes associated with metabolism, while gold circles represent the overexpression of acidic condition genes.

This study resulted in the development of the biomolecular model presented in Fig 4, which summarizes the conclusions from the observed differential gene expression data described above. This model explains how the observations from this experiment, where non-motile *E*. *coli* were anaerobically cultured in minimal liquid medium in space and on Earth, support the proposed reduced extracellular transport model as the primary cause of altered, *in vitro* bacterial behavior that has been observed to occur during spaceflight.

It was concluded that cells in space were under starvation conditions (circle 1 in Fig 4) based on the following observations: (a) the systematic overexpression of the *thiFGHS* genes (up to 32.41x), indicative of a lack of energy substrates in the extracellular environment [35]; (b) the overexpression of *dps* (up to 7.23x), which synthesizes a protein (Dps) that protects DNA molecules during starvation [43]; (c) the overexpression of *crp*, and *glnG* and *nac*, genes associated with carbon- and nitrogen-starvation, respectively, as well as the overexpression of *poxB*, one of the two genes associated with phosphate starvation [44]. We also observed the overexpression of metabolism genes not associated with glucose catabolism (glucose was the source of carbon in this experiment), indicated by circle 2. When substrate concentration decreases, cells devote more of their limited resources to the genome transcription needed to broaden the search for alternative sources of carbon, even if these are unavailable [38]. This was observed in the spaceflight samples by (a) the activation of mechanisms for oligopeptide transport (used by *E*. *coli* and *Salmonella typhimurium* as a source of carbon and energy [42]) through the cellular membrane, as shown by the overexpression of *malK* (up to 31.81x), *oppABCDF* (up to 5.88x), and *ompCF* (up to 9.84x). Another indication of this search for alternative sources of carbon was indicated by (b) the overexpression of the *malE* (up to 43.80-fold) and *lamB* (up to 30.05-fold) genes, responsible for the transport of maltose into the cell [40,41]. Future studies could include the exhaustive investigation of the transportases (and their associated genes) of all potential sources of carbon to specifically investigate this phenomenon. These responses are in line with what would be expected under reduced mass transport conditions where substrate uptake was limited to diffusion only.

Glucose catabolism genes were activated in space (circle 3), indicating an overall stimulation of metabolic activity—which could explain the observed increase in bacterial cell concentrations in microgravity: we quantified the overexpression of 69% of the genes known to us to control *E*. *coli*’s central Glucose metabolic pathways, and of 74% of the genes associated with metabolism but not directly involved in the glucose catabolism pathways. Stimulated metabolism and improved bacterial growth may sound counterintuitive to experiencing carbon starvation conditions. However, fed-batch processing (where nutrients are incrementally introduced) is shown to yield higher final cell concentrations (5- to 10-fold) than simple batch processing using the same total amount of substrate [54]. It had been previously proposed that a fed-batch-like condition also occurs in space where glucose uptake is diffusion limited, therefore resulting in similarly reduced nutrient availability in the local extracellular environment [29]. Furthermore, *E*. *coli* cultures were also shown to reach higher final cell concentrations in space while consuming the same amount of glucose as their matched ground controls [27]. Ref. [55] also reported an increase in final cell count in space and that the difference between spaceflight and Earth controls could be minimized by the addition of growth-limiting substrates. Collectively, the enhanced growth condition explanation suggested to occur as a result of the proposed altered environmental factors in microgravity is further supported by the new gene expression data.

We also observed the systematic overexpression of the *trpABCDE* operon (up to 69.11x), which is in charge of cellular amino acid biosynthesis and the subsequent formation of amino acids and organic acids [47,48]. Additionally, 88% of the genes associated with glucose catalysis into acetate were activated in the spaceflight cultures, indicating an increase in acetate production (circle 4), which is also of importance for biotechnology applications [1]. This increase in acetate and organic acids production could potentially lower the pH around the cell, which led us to the question if there were any indications that the cells experienced an increase in extracellular acidity. It did turn out that 78% of the genes induced by acetate but not by overall acidity were overexpressed in space (up to 10.50x) (circle 5). Additionally, from the 26 acetate- and overall acidity-induced genes presented in ref. [53], 23 were overexpressed in space (88%). These results again support that the bacterial cells in space experienced higher concentrations of acetate than their matched ground controls, and explain in part the decreased pH environment indicated by the activation of acid resistance mechanisms on the spaceflight cultures.

Similarly, 100% of the acid-response genes regulated by RpoS were overexpressed despite of the fact that *rpoS* was not up-regulated (actually, it was underexpressed in one of the three sets), serving as further indication of an increase in acidity in/around the cell, as these genes’ dependence on RpoS is reduced or even abolished under acid stress conditions [51]. Overall, from the 35 acid resistance-associated genes (Table 3), 28 (80%) were overexpressed in space (circle 6), further corroborating that, in space, the cells were living in an acidic environment. The genes that compose *E*. *coli*’s AR2 (*gadA/BC/E/W/X*), the most effective of its three acid resistance systems, were systematically overexpressed (100%) in space up to 25.60x (circle 7). Because AR2 requires glutamate, a molecule not provided in the growth medium, this finding also supports the hypothesis presented in ref. [36] that *E*. *coli* has a putative endogenous glutamate generator.

No statistically significant difference between the pH of the mixed bulk fluid of the space and Earth samples was observed. It is exactly this finding—no difference in the mixed fluid pH—that suggests the acid response exhibited by the cell was a result of the local build up, which would be sufficiently diluted when mixed so as to not be discernable. This is the basic premise of our conclusion as to why the individual cell would be affected and why this connection has been so challenging to assess prior to the gene expression data from this experiment.

The reduction in mass transport in space with respect to Earth may also explain the observed lack of trends in differential expression of most genes as a function of drug concentration (for example, see Table 1). While cultures in space were likely exposed to lower drug concentrations than their matched ground controls (similar to the glucose concentration gradient depicted in Fig 4), the difference between the spaceflight and Earth sets likely varied between the 25, 50 and 75 μg/mL groups, and it is reasonable to assume that this variation may not be linear. Future studies are needed to quantitatively assess the actual drug concentration that cells are exposed to in space, as this becomes important to inform drug dosages during long-duration space missions.

This new information also suggests how the spaceflight *in vitro* environment relates to conditions in the human body associated with bacterial virulence and mutation. In order to survive the 1.5<pH<2.5 mammalian gastric environment, bacteria usually need to be digested in large numbers (>10^9^) to survive. This is not the case, however, for *E*. *coli*, which can survive with as little as 100–1000 cells due to its effective acid resistance (AR) systems [49] (which is advantageous for a pathogen [56]). Once bacteria have survived exposure to the gastric environment, they eventually reach the colon, which is described as a being carbon-rich environment (equivalent to 40mM glucose), poor in phosphate (inorganic phosphate starvation can be considered as concentrations of 25 μM) and anoxic [44], forcing *E*. *coli* to enter stationary phase—especially when the cells are unattached to the large intestine’s mucosa [50]. Entering this stage shifts *E*. *coli*’s metabolism to fermentation, increasing the excretion of short-chain fatty acids, a raise in the concentration of acetic acid to 90 mM, and a decrease in pH to ~4.4 [44]. Although these conditions should be bactericidal, *E*. *coli* thrives, likely due to the *gad* genes, including *gadX*, which we saw systematically overexpressed in our spaceflight experiment (up to 13.73-fold). GadX, the protein synthesized by the *gadX* gene, is a regulator of virulence genes in enteropathogenic *E*. *coli* [57]. Furthermore, acidic conditions enhance the expression of numerous virulence factors, including the ToxR-ToxT virulence regulon in *Vibrio cholera*, the *phoP-phoQ* regulon of *Salmonella enterica*, and the pH 6 antigen of *Yersinia pestis* [58]. As such, the acid buildup around cells observed in space may also partly explain the observed increase in virulence of *Salmonella typhimurium* reported to occur during spaceflight [8]. This information also warrants further investigation on mutagenesis and mutation rate changes in space, since toxic metabolic products have been identified as a common cause of mutations [59] and it is now indicated that cells in space in liquid medium experience an extracellular metabolic byproduct buildup.

It had been previously reported that Hfq was a regulator of gene expression and increased virulence in space [8]. A comparative analysis of the 12 genes that exist in both the *Salmonella typhimurium* and *E*. *coli* databases that were differentially expressed in the experiment reported in Ref. [8], show that 11 (92%) match in differential expression trend (to be either over or underexpressed in space), as shown in S9 Table. However, our samples did not show an underexpression of *hfq*. Thus, it is recommended that future transcriptomic analyses of bacterial cultures grown in space include assessments of the Hfq regulon to provide further insight.

The altered extracellular environment model [27] hypothesizes that under conditions of diffusion-limited transport, the influx rate of nutrients from the bulk fluid into the cell will be reduced and similarly, a greater concentration of excreted byproducts will be present within the transport boundary layer of the cell. However, prior flight and ground experiments have not been able to directly assess these conditions and related ground experiments implementing physical measurement techniques (holographic interferometry) were unable to provide the necessary resolution for such small-scale fluid density gradient detection [32]. Similarly, computational model coefficients could not be sufficiently validated without more specific empirical data [22].

The observations made during our study allow us to conceptualize the series of events as follows: 1) the lack of gravity-driven sedimentation and buoyancy result in a substrate concentration gradient as the molecules consumed by the cell are replaced solely by Brownian motion from the bulk medium, therefore, nutrient uptake is limited to diffusion-only transport. This 2) stimulates the cell’s metabolism, as seen under similar fed-batch conditions on Earth, which in turn translates into 3) an increase in organic acid production. This increase, together with the lack of gravity driven forces and flows helping to remove the metabolic wastes, contribute to a 4) pH decrease locally around the cell.

The proposed altered extracellular transport model, is therefore now supported through gene expression data. This new information, along with prior related experimental data, strongly suggests that reduced extracellular mass transport is the primary underlying gravity-dependent, physical mechanism responsible, at least in part, for the altered microbial behavior typically reported to occur in space, including reduced lag phase duration, increased final population density, improved biofilm formation, higher specific productivity of secondary metabolites, modified cellular envelope, enhanced conjugation efficiency, increased virulence and reduced susceptibility to antibiotics. These unique observations could be further corroborated on future spaceflight experiments by using motile cells to serve as in flight controls for comparison to non-motile responses, by examining a variety of other bacterial species under differing growth conditions, and to acquire metabolite mass measurements to reach more generalized conclusions.

The microgravity environment is increasingly being utilized for myriad microbial applications, e.g. vaccine development, finding novel molecular targets against drug-resistant pathogens, and antibiotic production, as well as for numerous other non-bacterial investigations, e.g. on osteoporosis or cancer [60]. Knowledge gained from these bacterial studies and related lines of research aimed at elucidating how extracellular biophysical processes initiate mechanical transduction signals in cells in space can be applied to help address growing concerns of infectious disease outbreaks occurring worldwide and for protecting astronauts as they venture beyond Earth orbit.

## Materials and Methods

### Experiment Design

The data described in this manuscript was acquired from 24 FPAs (test tubes described below in Hardware) flown to the International Space Station (ISS) and 24 that remained as Earth controls. Each FPA had four chambers, separately containing the growth medium, bacterial inoculum, antibiotic, and fixative (described below in their respective sections). The driving scientific question of this experiment was about antibiotic effectiveness (published separately) and therefore samples contained Gentamicin Sulfate at different concentrations, as described in the Antibiotic section. Half of the FPAs contained a fixative and the other half a different one, as described in the Fixative section. Each tested condition was flown in quadruplicates, thus the 3 concentrations in two different fixatives accounted for 24 FPAs. Sixteen spaceflight and 16 Earth FPAs contained samples with no antibiotics, but were lamentably requested to be fixed too early in the experiment to be used in this analysis. *n = 4* for each tested condition with a few exceptions, where samples were not taken into account because not the entire antibiotic solution was introduced into the growth chamber. Samples fixed with PFA: all were *n = 4* except for spaceflight at 25 and 50 μg/ml (*n = 3*, each). Samples fixed with RNA Later II: *n = 4* in all cases with the exception of Space and Earth 50 μg/ml, and Space 75 μg/ml, which had *n = 3*.

### Bacterial model

Non-pathogenic *E*. *coli* ATCC 4157 *Escherichia coli* (Migula) Castellani and Chalmers (ATCC^®^ 4157^™^) was chosen as the model species and strain for myriad reasons. *E*. *coli* is the bacterial research organism flown to space the most, which provides a wealth of data to compare against [61]. Specifically, this strain has been flown in seven different space shuttle flights: STS-37, -43, -50, -54, -57, -60 and -62 [20,27]. This is a non-motile strain when grown with glucose as the source of carbon. This is important because motile cells could potentially disrupt the proposed quiescent local fluid environment, which in turn may eradicate spaceflight effects [31]. As part of the human biome, *E*. *coli* will naturally be present in all human spaceflights and has been found on spacecraft surfaces and air [62]. It is an opportunistic pathogen, so has the potential to trigger infectious diseases during spaceflight [63], *e*.*g*. meningitis, invasive urinary tract infections, septicemia and diarrhea [64,65]. Finally, *E*. *coli* is an organism commonly used for genetic engineering studies, thus acquiring more knowledge about how it responds to the novel environment of space may prove beneficial to other applications.

### Growth medium and temperature

*E*. *coli* was grown anaerobically in Medium E minimal medium as described in ref. [66] supplemented with glucose (Fisher Scientific, Cat. No. D-16, Waltham, MA, USA) to a final $5\frac{g}{L}$ concentration (27.8 mM). To replicate the temperature changes that took place on orbit during operations, ground controls were stored and operated inside BioServe’s environmental test chamber to mimic the ISS humidity and temperature profiles. The experiment was designed for bacteria to be cultured at 30°C, as this temperature allows for a clearer differentiation of the growth phases [67]. Recorded temperature data show that the spaceflight samples were maintained at 30.2°C ±0.7°C (from four independent sensors) and the ground controls at 31.7°C ±0.4°C (from another four independent sensors), thus there was a 1.5°C average temperature difference. The inoculum’s cell density was $7.91\times 10^{6}\frac{cell}{mL}$ (log-phase cells), which once diluted to the test starting conditions yielded a $1.22\times 10^{6}\frac{cell}{mL}$ concentration.

### Antibiotics

The antibiotic used was Gentamicin Sulfate (MP Biomedical, Cat No. 1676045, Santa Ana, CA, USA), an aminoglycoside that interrupts protein synthesis by binding to the 30S subunit of the bacterial ribosome and which was previously flown in experiments onboard STS-69 and STS-73 [67] and the Soviet/Russian space station Mir [68] (reported in the last reference simply as Gentamicin, with no further details). Gentamicin Sulfate solutions were prepared in distilled water and filter-sterilized (0.20 *μm*) for flight. Their concentrations varied so that, when introduced into the culture, they would range from 25 to $75\;\frac{\mu g}{mL}$. They were stored at 4°C until needed for loading the FPAs (see Hardware, Sample Preparation and Loading, and Operations Timeline).

### Fixative

Since all analyses were done post-flight, the samples were fixed on orbit in paraformaldehyde (PFA) for the phenotypic studies and in RNA Later II for the genotypic assays. Paraformaldehyde (ACROS, Cat. No. 41678, New Jersey, USA) solutions in PBS (Fisher Scientific, Cat. No. TA-125-PB, Waltham, MA, USA) were prepared (pH 7.0) and filter sterilized (0.20 *μm*) so that, when mixed with the cultures, would yield a 1.5% concentration. RNA Later II (Life Technologies, Cat No. B7024, Carlsbad, CA, USA) at a 0.6 fixative/sample volume ratio was used for the gene expression analysis samples. Samples were fixed during stationary phase.

### Hardware

**Fig 5:**
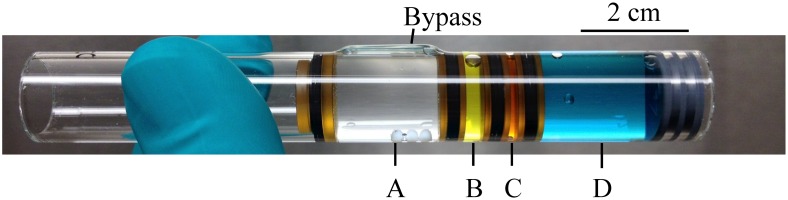
Fluid Processing Apparatus (FPA). BioServe’s Fluid Processing Apparatus (FPA) loaded with colored solutions to best describe the actual contents per chamber of the AES-1 configuration: A– 2.75 mL of sterile growth medium with glucose; B– 0.50 mL of inoculum in minimal medium without glucose; C– 0.25 mL of antibiotic solution; D– 2.10 mL of fixative (either paraformaldehyde or RNA Later II). In this figure, pushing from right to left would move the septum separating chambers A and B into the bypass, thus allowing for the solution in chamber B to be transferred and mixed into chamber A, and likewise to later add the contents of C and D. The actual solutions as flown in AES-1 were all colorless.

The experiments took place in BioServe’s Fluid Processing Apparatus (FPA), shown in Fig 5. The FPA is a spaceflight-rated glass barrel that can store four different solutions for up to a combined total of 6.5 mL and then mix them sequentially to initiate and terminate an experiment. Fluids are separated by septa, which can be pushed to allow mixing through a bypass. FPAs were packed in groups of eight inside Group Activation Packs (GAP), which in turn were housed inside BioServe’s Commercial Generic Bioprocessing Apparatus (CGBA) for temperature control [69].

### Sample Preparation and Loading

All of the hardware items were autoclave-sterilized. Two sterile PTFE mixing balls were introduced together with 2.75 mL of sterile Medium E with $5.91\frac{g}{L}$ glucose (to yield a final $5\frac{g}{L}$ (27.8 mM) concentration when mixed with the inoculum) into the A chamber of each FPA. The FPAs were then incubated for 48 hours at 37°C for contamination check. Next, chamber B was loaded with 0.50 of inoculum (at $7.90\times 10^{6}\;\frac{cell}{mL}$ to yield a $1.22\times 10^{6}\;\frac{cell}{mL}$ concentration when mixed with the growth medium) in glucose-free Medium E to limit growth before activation. Chamber C was then loaded with 0.25 mL of antibiotic solution as necessary for each experimental condition. Finally, the corresponding fixative was introduced into Chamber D (2.10 mL). The FPAs were stored at 4°C and transported from Boulder, CO to NASA Wallops in Virginia (both flight and ground controls). There, the RNA Later II solution was re-homogenized since this fixative tends to form crystals at lower temperatures. The FPAs were then loaded into GAPs and these, in turn, into CGBA where they were maintained at 4°C from payload handover until experimentation activation. The ground controls were similarly prepared and maintained.

### Operations Timeline

**Fig 6:**
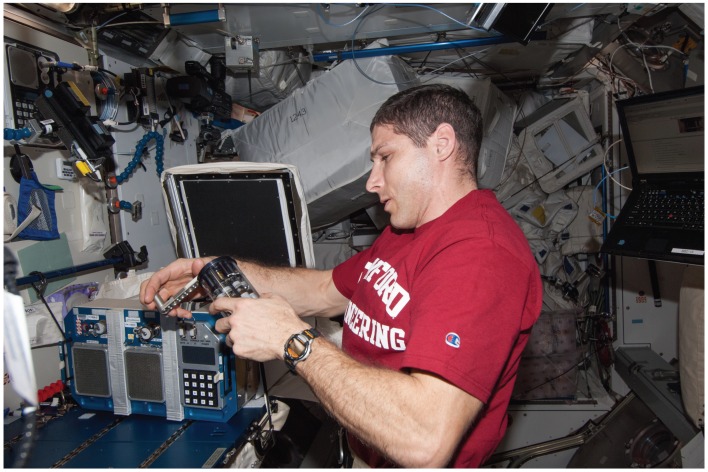
AES-1 operation in space. Astronaut Mike Hopkins operating one of the GAPs containing the AES-1 experiment onboard ISS (Photo credit: NASA). The individual in this manuscript has given written informed consent (as outlined in PLOS consent form) to publish these case details.

Antibiotic solutions were prepared on December 10, 2014 and were maintained at 4°C afterwards. The loading of the hardware was finalized and it was handed over for integration into the Cygnus spacecraft on December 13, 2014. The experiment hardware was maintained at 4°C until experiment start. AES-1 launched onboard Orbital CRS-1 on January 9, 2014 with the samples held at 4°C. After being berthed to ISS, three days later the samples were transferred from the transport CGBA to another CGBA onboard Station waiting at 4°C. After transfer, the ISS CGBA was commanded to 30°C and 23 hours later, the first activation took place (introduction of the inoculum into the growth medium, as seen in Fig 6). Nineteen hours later the second activation was conducted (introduction of the antibiotic solution into the growth chamber). Thirty hours later the experiment was terminated (fixative allowed to mix with the culture). Thus, the experiment total processing time was 49 hours. After the experiment conclusion, the GAPs containing FPAs with PFA as fixative were stored in the ISS CGBA, which was then commanded to 20°C. The ones with RNA Later II were placed in the MELFI freezer at -75°C. The space samples remained stored as indicated until their return to Earth. The PFA-fixed samples came back on SpaceX-3, which landed May 18, 2015, and the RNA Later II samples returned on SpaceX-4 on October 25, 2015. All of these operations were repeated on Earth for the ground controls with the same timing and conditions, but with an 8 hour offset. Specific dates and times for each of the operational steps are provided in Appendix 2 of Zea (2015).

### Optical Density, Cell Count and pH

All of the analyses were conducted in labs post-flight after sample return to Earth. Optical Density (OD) measurements of the FPAs fixed with PFA were acquired with a Fisher Scientific Multiskan^™^ FC Microplate Photometer at 600 nm. Three individual samples were taken from each FPA, thus totaling over 500 OD data points. Due to the significant change in cell size on the spaceflight samples (published separately), the OD data was determined to be inaccurate; therefore, actual cell counts using a hemacytometer were conducted on each of the samples. pH measurements were taken of the fixed (in PFA) samples after briefly vortexing. Thus, measurements were taken of the mixed bulk fluid, taking into account the zones immediate to the cells as well as the overall bulk fluid. Measurements were taken with a Thermo Scientific Orion 9810BN Micro pH Electrode at ~25°C.

### RNA-seq library preparation, sequencing and data analysis

Four ml of the bacterial suspension in RNALater II and Medium E growth medium were spun down at 200 g’s for 5 min. The supernatant was discarded and the pellet were resuspended in 200 μl PBS and mixed by pipetting. 100 μl aliquots were taken out for DNA and RNA extraction from the same sample. RNA extraction was done using Qiagen RNeasy mini kit (Qiagen, Hilden, Germany) with on-column DNase digestion. Final elution was made in 30 μL dH_2_O. The concentration and integrity of the total RNA was estimated by Quant-iT^™^ RiboGreen^®^ RNA Assay Kit (ThermoFisher Scientific, Waltham, MA), and Agilent 2100 Bioanalyzer (Applied Biosystems, Carlsbad, CA), respectively. Ribosomal RNA (rRNA) was removed using Ribo-Zero^™^ Gold (Yeast) kit (Epicenter, Madison, WI) using manufacturer's recommended protocol. Immediately after the rRNA removal the RNA was fragmented and primed for the first strand synthesis using the NEBnext First Strand synthesis module (New England BioLabs Inc., Ipswich, MA). The second strand synthesis was then performed using the NEBnext Second Strand synthesis module. Following this the samples were taken into standard library preparation protocol using NEBNext^®^ DNA Library Prep Master Mix Set for Illumina^®^ with slight modifications. Briefly, end-repair was done followed by polyA addition and adapter ligation. Post-ligated materials were individually barcoded with primers and amplified through 12 cycles of PCR. Final library quantity was assessed by Quant-iT^™^ PicoGreen^®^ dsDNA Assay Kit and the library quality was estimated on LabChip^®^ GX (Caliper, PerkinElmer, Waltham, MA). Accurate quantification of the final libraries for sequencing applications was determined using the qPCR-based KAPA Biosystems Library Quantification kit (Kapa Biosystems, Inc., Woburn, MA). Each library was diluted to a final concentration of 12.5 nM and pooled equimolar prior to clustering. Paired-End (PE) sequencing on an Illumina HiSeq2500 sequencer (Illumina, Inc.). Image analysis and base calling was performed using the standard Illumina Pipeline consisting of Real Time Analysis (RTA) version v1.18.64. Raw reads were de-multiplexed using a bcl2fastq conversion software v1.8.3 (Illumina, Inc.) with default settings. Post-processing of the sequencing reads from RNA-seq experiments for each sample was performed using HudsonAlpha’s unique in-house RNA-seq data analysis pipeline. Briefly, quality control checks on raw sequence data for each sample were performed using FastQC (Babraham Bioinformatics, Cambridge, UK). Data-analysis was performed using the CLC Genomics Workbench (Version 7.5.1, CLC Bio, Aarhus, Denmark). The reference genome *Escherichia coli* (DH10B) sequence was downloaded from the UCSC genome browser. For read mapping, the following parameters were used: mismatch cost = 2, insertion and deletion cost = 3, length fraction: 0.8, similarity fraction = 0.8, global alignment = no, auto-detect paired distances = yes. Samples were grouped and differential expression of genes was calculated on the basis of fold changes (using the default cut-off ≥ ±2.0) observed in comparisons between defined conditions. RNA sequencing statistical data is described in S10 Table. The Spearman correlation (Fig 1) was generated using R software (v 3.2.4). Gene set analysis was performed using the PANTHER over representation test (release 2016-07-15) for the candidate genes Tables 1, 2, and 3. The candidate genes were analyzed using the Gene Ontology Database (release 2016-08-22). The results are presented as S11–S13 Tables.

## Supporting Information

S1 Table Differentially expressed genes per sets. Number of genes that were differentially expressed in space, with respect to their matched Earth (1g) controls (from over 4,000 genes in the *E*. *coli* (DH10B) sequence). The only two commonly under-expressed genes were *hokE* and *rzoD*.
(DOCX)

S2 Table Overexpressed genes in all three sets in space. S1 Table. Fold-increase of the genes that were overexpressed in space in all the three testing conditions, with respect to their matched Earth (1g) controls.
(DOCX)

S3 Table Over 10x overexpression in space– 25 μg/mL set. Fold-increase of the genes that were overexpressed in space at least a 10-fold, with respect to their matched Earth (1g) controls, in the 25 μg/mL set.
(DOCX)

S4 Table Over 10x overexpression in space– 50 μg/mL set. Fold-increase of the genes that were overexpressed in space at least a 10-fold, with respect to their matched Earth (1g) controls, in the 50 μg/mL set.
(DOCX)

S5 Table Over 10x overexpression in space– 75 μg/mL set. Fold-increase of the genes that were overexpressed in space at least a 10-fold, with respect to their matched Earth (1g) controls, in the 75 μg/mL set.
(DOCX)

S6 Table Non-glucose catabolism metabolism genes. Differential expression of genes associated with metabolism but not directly involved in the glucose catabolism pathways analyzed in Fig 3. List compiled from ref. [45], [46]. The last three columns are graphical indicators of non-differential expression (white cells), over- (black cells) and under-expression (diagonal lines cells).
(DOCX)

S7 Table Acetate production from glucose genes. Differential expression of genes associated with acetate production from glucose. List compiled from ref. [45], [46]. The last three columns are graphical indicators of non-differential expression (white cells), over- (black cells) and under-expression (diagonal lines cells).
(DOCX)

S8 Table Genes induced by acetate but not by acidity. Differential expression of genes induced by acetate but not by acidity (comparing the lists from ref. [53] of acetate-induced genes, and ref. [36] list of genes induced by acidity). The last three columns are graphical indicators of non-differential expression (white cells), over- (black cells) and under-expression (diagonal lines cells).
(DOCX)

S9 Table Hfq Regulon. Comparative analysis of the 12 genes that exist in both the *Salmonella typhimurium* and *E*. *coli* databases that were differentially expressed in the experiment reported in Ref. [8]. Eleven (92%) match in differential expression trend (to be either over or underexpressed in space). *hfq* was underexpressed (-3.36x) in Ref. [8] but non-differentially expressed in the 25 and 75 μg/mL sets, and overexpressed (3.20x) in the 50 μg/mL group.
(DOCX)

S10 Table Sequencing Matrix. RNA sequencing statistical data. The cut off for adjusted p-value was 0.05 with FDR correction of 0.05.
(DOCX)

S11 Table Gene set analysis on differentially expressed genes from Table 1. Gene set analysis was performed using the PANTHER over representation test (release 2016-07-15) for the candidate genes in Table 1. The candidate genes were analyzed using the Gene Ontology Database (release 2016-08-22) (GORGP, 2015).
(DOCX)

S12 Table Gene set analysis on differentially expressed genes from Table 2. Gene set analysis was performed using the PANTHER over representation test (release 2016-07-15) for the candidate genes in Table 2. The candidate genes were analyzed using the Gene Ontology Database (release 2016-08-22) (GORGP, 2015).
(DOCX)

S13 Table Gene set analysis son differentially expressed genes from Table 3. Gene set analysis was performed using the PANTHER over representation test (release 2016-07-15) for the candidate genes in Table 3. The candidate genes were analyzed using the Gene Ontology Database (release 2016-08-22) (GORGP, 2015).
(DOCX)
