# Supplementary material for: A Molecular Genetic Basis Explaining Altered Bacterial Behavior in Space
Source: PLoS One. 2016 Nov 2;11(11):e0164359. doi: 10.1371/journal.pone.0164359 (PMC5091764; doi:10.1371/journal.pone.0164359)
Supplement: S1 Table — Number of genes that were differentially expressed in space, with respect to their matched Earth (1g) controls (from over 4,000 genes in the E. coli (DH10B) sequence). The only two commonly under-expressed genes were hokE and rzoD. (DOCX) [file pone.0164359.s001.docx]

**S1 Table. Differentially expressed genes per sets.** Number of genes that were differentially expressed in space, with respect to their matched Earth (1g) controls (from over 4,000 genes in the *E. coli* (DH10B) sequence). The only two commonly under-expressed genes were *hokE* and *rzoD*.

|  | 25 µg/mL | 50 µg/mL | 75 µg/mL | In common |
| --- | --- | --- | --- | --- |
| Underexpressed | 1587 | 30 | 50 | 2 |
| Overexpressed | 270 | 2213 | 782 | 81 |
