# Supplementary material for: A Molecular Genetic Basis Explaining Altered Bacterial Behavior in Space
Source: PLoS One. 2016 Nov 2;11(11):e0164359. doi: 10.1371/journal.pone.0164359 (PMC5091764; doi:10.1371/journal.pone.0164359)
Supplement: S2 Table — S1 Table. Fold-increase of the genes that were overexpressed in space in all the three testing conditions, with respect to their matched Earth (1g) controls. (DOCX) [file pone.0164359.s002.docx]

**S2 Table. Overexpressed genes in all three sets in space.** Fold-increase of the genes that were overexpressed in space in all the three testing conditions, with respect to their matched Earth (1g) controls.

| Gene | 25 µg/mL | 50 µg/mL | 75 µg/mL |
| --- | --- | --- | --- |
| *acnA* | 2.22 | 2.72 | 2.17 |
| *agp* | 3.19 | 3.42 | 2.89 |
| *asnB* | 2.40 | 3.98 | 3.30 |
| *bfr* | 5.32 | 8.19 | 3.93 |
| *bolA* | 2.59 | 3.21 | 2.44 |
| *elaB* | 4.48 | 4.20 | 2.11 |
| *entS_2* | 2.36 | 14.44 | 2.45 |
| *fbaA* | 2.19 | 4.72 | 2.65 |
| *fbaB* | 4.18 | 5.77 | 2.36 |
| *fepD_1* | 2.09 | 6.73 | 2.43 |
| *fimZ* | 2.28 | 4.38 | 2.60 |
| *gadW* | 8.33 | 5.69 | 2.25 |
| *gadX* | 13.73 | 5.82 | 2.05 |
| *gltA* | 2.56 | 4.64 | 4.32 |
| *hdeA* | 6.24 | 28.09 | 2.66 |
| *hdeB* | 6.10 | 29.08 | 2.68 |
| *hdeD* | 3.04 | 14.86 | 2.98 |
| *hdhA* | 2.89 | 2.47 | 2.02 |
| *ibpB* | 5.86 | 3.49 | 2.35 |
| *iscU* | 2.09 | 2.93 | 2.43 |
| *lpp* | 3.63 | 2.98 | 2.11 |
| *mfd* | 2.99 | 3.49 | 2.08 |
| *mscL* | 2.48 | 4.48 | 2.41 |
| *mtlA* | 3.56 | 3.59 | 2.01 |
| *narG* | 2.09 | 4.85 | 2.17 |
| *narJ* | 3.39 | 3.56 | 2.01 |
| *oppC* | 2.06 | 4.30 | 3.33 |
| *oppD* | 2.69 | 4.27 | 3.45 |
| *oppF* | 2.52 | 4.77 | 3.63 |
| *phoH* | 2.55 | 2.73 | 2.79 |
| *psiF* | 4.85 | 2.10 | 2.25 |
| *rmf* | 3.87 | 3.96 | 2.07 |
| *sdhA* | 2.01 | 2.12 | 2.10 |
| *sdhB* | 2.16 | 2.15 | 2.31 |
| *sra* | 7.33 | 6.28 | 2.53 |
| *sucA* | 3.37 | 4.07 | 2.96 |
| *sucB* | 4.29 | 3.60 | 2.84 |
| *sucC* | 3.59 | 4.16 | 3.11 |
| *sucD* | 4.35 | 4.39 | 3.18 |
| *sufA* | 7.05 | 5.08 | 2.11 |
| *talA* | 6.08 | 4.77 | 2.18 |
| *thiE* | 2.40 | 28.59 | 6.57 |
| *thiF* | 2.41 | 28.87 | 7.44 |
| *thiG* | 2.26 | 30.48 | 8.04 |
| *thiH* | 2.20 | 24.88 | 7.44 |
| *thiS* | 2.49 | 32.41 | 6.01 |
| *ybbW* | 2.04 | 3.14 | 2.42 |
| *ybeY* | 2.83 | 3.34 | 2.10 |
| *ybeZ* | 2.64 | 3.81 | 2.15 |
| *ybhP* | 3.78 | 6.45 | 2.02 |
| *ybiM* | 4.58 | 2.40 | 2.08 |
| *ycaC* | 6.97 | 5.92 | 2.42 |
| *yccJ* | 28.70 | 7.22 | 2.13 |
| *ycgB* | 5.82 | 3.93 | 2.48 |
| *ycgX* | 2.00 | 2.52 | 2.39 |
| *yciF* | 2.52 | 3.37 | 3.50 |
| *ydcH* | 2.80 | 4.82 | 2.09 |
| *ydcK* | 2.22 | 3.44 | 2.19 |
| *ydiZ* | 3.12 | 4.58 | 3.85 |
| *yeaG* | 4.31 | 4.73 | 3.29 |
| *yeaH* | 3.39 | 3.74 | 2.29 |
| *yeaQ* | 9.95 | 6.26 | 3.80 |
| *yegP* | 23.93 | 5.91 | 2.21 |
| *yehE* | 2.82 | 3.25 | 4.77 |
| *ygjG* | 7.14 | 3.76 | 2.22 |
| *yhaH* | 3.46 | 3.62 | 2.30 |
| *yhcH* | 2.70 | 4.44 | 2.17 |
| *yhiD* | 6.67 | 19.57 | 2.16 |
| *yhiF* | 6.78 | 6.29 | 2.36 |
| *yhiJ* | 2.72 | 3.67 | 2.38 |
| *yhiM* | 6.91 | 6.94 | 2.10 |
| *yiaG* | 14.22 | 8.45 | 4.36 |
| *yjbE* | 4.17 | 5.62 | 5.44 |
| *yjbM* | 4.91 | 3.78 | 2.31 |
| *yjdI* | 6.26 | 12.76 | 3.06 |
| *yjgL* | 2.81 | 4.43 | 2.12 |
| *ykgC* | 2.78 | 8.13 | 5.60 |
| *ykgH* | 2.05 | 7.17 | 2.16 |
| *yncG* | 3.33 | 5.35 | 2.34 |
| *yqeK* | 2.74 | 4.20 | 5.25 |
| *ysgA* | 2.08 | 3.77 | 2.99 |
