# Supplementary material for: A Molecular Genetic Basis Explaining Altered Bacterial Behavior in Space
Source: PLoS One. 2016 Nov 2;11(11):e0164359. doi: 10.1371/journal.pone.0164359 (PMC5091764; doi:10.1371/journal.pone.0164359)
Supplement: S3 Table — Fold-increase of the genes that were overexpressed in space at least a 10-fold, with respect to their matched Earth (1g) controls, in the 25 μg/mL set. (DOCX) [file pone.0164359.s003.docx]

**S3 Table. Over 10x overexpression in space – 25 µg/mL set.**  Fold-increase of the genes that were overexpressed in space at least a 10-fold, with respect to their matched Earth (1g) controls, in the 25 µg/mL set.

| Gene | Fold increase |
| --- | --- |
| *yccJ* | 28.70 |
| *wrbA* | 24.76 |
| *ecnB* | 24.64 |
| *yegP* | 23.93 |
| *gadE* | 23.47 |
| *yhcO* | 20.17 |
| *hlyE* | 18.90 |
| *hyaC* | 18.25 |
| *ygeM* | 15.80 |
| *hyaD* | 15.54 |
| *slp* | 14.78 |
| *yiaG* | 14.22 |
| *gadC* | 14.18 |
| *gadA* | 14.07 |
| *gadX* | 13.73 |
| *gadB* | 12.22 |
| *hyaB* | 12.00 |
| *yohC* | 11.70 |
| *hyaF* | 11.08 |
