# Supplementary material for: A Molecular Genetic Basis Explaining Altered Bacterial Behavior in Space
Source: PLoS One. 2016 Nov 2;11(11):e0164359. doi: 10.1371/journal.pone.0164359 (PMC5091764; doi:10.1371/journal.pone.0164359)
Supplement: S4 Table — Fold-increase of the genes that were overexpressed in space at least a 10-fold, with respect to their matched Earth (1g) controls, in the 50 μg/mL set. (DOCX) [file pone.0164359.s004.docx]

**S4 Table. Over 10x overexpression in space – 50 µg/mL set.**  Fold-increase of the genes that were overexpressed in space at least a 10-fold, with respect to their matched Earth (1g) controls, in the 50 µg/mL set.

| Gene | Fold increase |
| --- | --- |
| *trpA* | 69.11 |
| *trpD* | 60.78 |
| *trpB* | 53.43 |
| *trpE* | 46.74 |
| *trpC* | 45.61 |
| *malE* | 43.80 |
| *thiS* | 32.41 |
| *thiG* | 30.48 |
| *hdeB* | 29.08 |
| *thiF* | 28.87 |
| *thiE* | 28.59 |
| *yiaW* | 28.55 |
| *hdeA* | 28.09 |
| *gadB* | 25.60 |
| *ompL* | 24.88 |
| *thiH* | 24.88 |
| *thiC* | 24.67 |
| *yfbN* | 24.38 |
| *lamB* | 23.63 |
| *gadE* | 23.04 |
| *malK* | 22.39 |
| *yhjX* | 21.96 |
| *yhiD* | 19.57 |
| *gadA* | 17.49 |
| *ygeI* | 17.30 |
| *entC_1* | 15.22 |
| *entC_2* | 14.94 |
| *hdeD* | 14.86 |
| *yjgI* | 14.85 |
| *entS_2* | 14.44 |
| *yqeH* | 14.27 |
| *entS_1* | 13.82 |
| *flgB* | 13.03 |
| *yjdI* | 12.76 |
| *fes_2* | 12.27 |
| *yfdS* | 11.52 |
| *yqeI* | 11.39 |
| *fepA_2* | 11.29 |
| *gadC* | 11.11 |
| *yhdU* | 10.62 |
| *metA* | 10.50 |
| *bioB* | 10.12 |
| *hmp* | 10.02 |
