# Supplementary material for: A Molecular Genetic Basis Explaining Altered Bacterial Behavior in Space
Source: PLoS One. 2016 Nov 2;11(11):e0164359. doi: 10.1371/journal.pone.0164359 (PMC5091764; doi:10.1371/journal.pone.0164359)
Supplement: S5 Table — Fold-increase of the genes that were overexpressed in space at least a 10-fold, with respect to their matched Earth (1g) controls, in the 75 μg/mL set. (DOCX) [file pone.0164359.s005.docx]

**S5 Table. Over 10x overexpression in space – 75 µg/mL set.** Fold-increase of the genes that were overexpressed in space at least a 10-fold, with respect to their matched Earth (1g) controls, in the 75 µg/mL set.

| Gene | Fold increase |
| --- | --- |
| *malE* | 36.07 |
| *malK* | 31.81 |
| *trpE* | 30.14 |
| *lamB* | 30.05 |
| *trpD* | 26.28 |
| *trpC* | 19.13 |
| *trpB* | 18.84 |
| *trpA* | 18.74 |
| *flgB* | 12.86 |
| *flgC* | 12.05 |
| *flgE* | 11.98 |
| *flgD* | 10.05 |
