# Supplementary material for: A Molecular Genetic Basis Explaining Altered Bacterial Behavior in Space
Source: PLoS One. 2016 Nov 2;11(11):e0164359. doi: 10.1371/journal.pone.0164359 (PMC5091764; doi:10.1371/journal.pone.0164359)
Supplement: S6 Table — Differential expression of genes associated with metabolism but not directly involved in the glucose catabolism pathways analyzed in Fig 3. List compiled from ref. [45], [46]. The last three columns are graphical indicators of non-differential expression (white cells), over- (black cells) and under-expression (diagonal lines cells). (DOCX) [file pone.0164359.s006.docx]

**S6 Table. Non-glucose catabolism metabolism genes.** Differential expression of genes associated with metabolism but not directly involved in the glucose catabolism pathways analyzed in Figure 3. List compiled from ref. 45, 46. The last three columns are graphical indicators of non-differential expression (white cells), over- (black cells) and under-expression (diagonal lines cells).

| Gene name | 25 μg/mL | 50 μg/mL | 75 μg/mL | 25 | 50 | 75 |
| --- | --- | --- | --- | --- | --- | --- |
| *cusF_1* | -1.89 | -3.75 | -2.27 |  |  |  |
| *cusF_2* | -6.90 | 1.99 | -3.38 |  |  |  |
| *malE* | -1.36 | 43.80 | 36.07 |  |  |  |
| *malK* | -1.58 | 22.39 | 31.81 |  |  |  |
| *oppA* | 1.11 | 5.87 | 3.84 |  |  |  |
| *oppB* | 1.78 | 4.68 | 3.45 |  |  |  |
| *oppC* | 2.06 | 4.30 | 3.33 |  |  |  |
| *oppD* | 2.69 | 4.27 | 3.45 |  |  |  |
| *oppF* | 2.52 | 4.77 | 3.63 |  |  |  |
| *poxB* | 8.77 | 3.48 | 1.79 |  |  |  |
| *sucA* | 3.37 | 4.07 | 2.96 |  |  |  |
| *sucB* | 4.29 | 3.60 | 2.84 |  |  |  |
| *sucC* | 3.59 | 4.16 | 3.11 |  |  |  |
| *sucD* | 4.35 | 4.39 | 3.18 |  |  |  |
| *thiE* | 2.40 | 28.59 | 6.57 |  |  |  |
| *thiF* | 2.41 | 28.87 | 7.44 |  |  |  |
| *thiG* | 2.26 | 30.48 | 8.04 |  |  |  |
| *thiH* | 2.20 | 24.88 | 7.44 |  |  |  |
| *thiS* | 2.49 | 32.41 | 6.01 |  |  |  |
| *trpA* | -5.10 | 69.11 | 18.74 |  |  |  |
| *trpB* | -5.20 | 53.43 | 18.84 |  |  |  |
| *trpC* | -6.65 | 45.61 | 19.13 |  |  |  |
| *trpD* | -6.85 | 60.78 | 26.28 |  |  |  |
| *trpE* | -8.63 | 46.74 | 30.14 |  |  |  |
