# Supplementary material for: A Molecular Genetic Basis Explaining Altered Bacterial Behavior in Space
Source: PLoS One. 2016 Nov 2;11(11):e0164359. doi: 10.1371/journal.pone.0164359 (PMC5091764; doi:10.1371/journal.pone.0164359)
Supplement: S7 Table — Differential expression of genes associated with acetate production from glucose. List compiled from ref. [45], [46]. The last three columns are graphical indicators of non-differential expression (white cells), over- (black cells) and under-expression (diagonal lines cells). (DOCX) [file pone.0164359.s007.docx]

**S7 Table. Acetate production from glucose genes.** Differential expression of genes associated with acetate production from glucose. List compiled from ref. 45, 46. The last three columns are graphical indicators of non-differential expression (white cells), over- (black cells) and under-expression (diagonal lines cells).

| Gene  Name | 25 μg/mL | 50 μg/mL | 75 μg/mL | 25 | 50 | 75 |
| --- | --- | --- | --- | --- | --- | --- |
| *aceE* | -1.69 | 3.42 | 2.35 |  |  |  |
| *aceF* | -1.27 | 3.57 | 2.52 |  |  |  |
| *lpd* | -1.20 | 2.91 | 2.20 |  |  |  |
| *pflB* | -1.06 | 5.07 | 4.04 |  |  |  |
| *poxB* | 8.77 | 3.48 | 1.79 |  |  |  |
| *ptsG* | -2.06 | 2.41 | 1.49 |  |  |  |
| *pykA* | -1.32 | 3.38 | 1.83 |  |  |  |
| *pykF* | -1.62 | 3.61 | 2.02 |  |  |  |
