# Supplementary material for: A Molecular Genetic Basis Explaining Altered Bacterial Behavior in Space
Source: PLoS One. 2016 Nov 2;11(11):e0164359. doi: 10.1371/journal.pone.0164359 (PMC5091764; doi:10.1371/journal.pone.0164359)
Supplement: S8 Table — Differential expression of genes induced by acetate but not by acidity (comparing the lists from ref. [53] of acetate-induced genes, and ref. [36] list of genes induced by acidity). The last three columns are graphical indicators of non-differential expression (white cells), over- (black cells) and under-expression (diagonal lines cells). (DOCX) [file pone.0164359.s008.docx]

**S8 Table. Genes induced by acetate but not by acidity.** Differential expression of genes induced by acetate but not by acidity (comparing the lists from ref. 53 of acetate-induced genes, and ref. 36 list of genes induced by acidity). The last three columns are graphical indicators of non-differential expression (white cells), over- (black cells) and under-expression (diagonal lines cells).

| Gene  Name | 25 μg/mL | 50 μg/mL | 75 μg/mL | 25 | 50 | 75 |
| --- | --- | --- | --- | --- | --- | --- |
| *adhE* | 1.85 | 5.73 | 3.47 |  |  |  |
| *katE* | 1.28 | 2.43 | 1.71 |  |  |  |
| *aslB* | -3.16 | 1.45 | 1.61 |  |  |  |
| *fbaB* | 4.18 | 5.77 | 2.36 |  |  |  |
| *glnK* | -1.31 | 4.15 | 2.56 |  |  |  |
| *grxB* | -1.33 | 4.06 | 2.89 |  |  |  |
| *metA* | -4.55 | 10.50 | 4.80 |  |  |  |
| *pflB* | -1.06 | 5.07 | 4.04 |  |  |  |
| *talA* | 6.08 | 4.77 | 2.18 |  |  |  |
