# Supplementary material for: A Molecular Genetic Basis Explaining Altered Bacterial Behavior in Space
Source: PLoS One. 2016 Nov 2;11(11):e0164359. doi: 10.1371/journal.pone.0164359 (PMC5091764; doi:10.1371/journal.pone.0164359)
Supplement: S9 Table — Comparative analysis of the 12 genes that exist in both the Salmonella typhimurium and E. coli databases that were differentially expressed in the experiment reported in Ref. [8]. Eleven (92%) match in differential expression trend (to be either over or underexpressed in space). hfq was underexpressed (-3.36x) in Ref. [8] but non-differentially expressed in the 25 and 75 μg/mL sets, and overexpressed (3.20x) in the 50 μg/mL group. (DOCX) [file pone.0164359.s009.docx]

**S9 Table. Hfq Regulon.** Comparative analysis of the 12 genes that exist in both the *Salmonella typhimurium* and *E. coli* databases that were differentially expressed in the experiment reported in Ref. 8. Eleven (92%) match in differential expression trend (to be either over or underexpressed in space). *hfq* was underexpressed (-3.36x) in Ref. 8 but non-differentially expressed in the 25 and 75 µg/mL sets, and overexpressed (3.20x) in the 50 µg/mL group.

| Gene  Name | Ref. 8 | 25 μg/mL | 50 μg/mL | 75 μg/mL |
| --- | --- | --- | --- | --- |
| *ompA* | 2.05 | 1.35 | 2.93 | 1.70 |
| *ompC* | 2.44 | -1.05 | 2.80 | 1.33 |
| *gapA* | 7.67 | 1.59 | 5.35 | 2.77 |
| *adhE* | 4.75 | 1.85 | 5.73 | 3.47 |
| *glpQ* | 2.58 | -1.06 | 2.14 | 1.57 |
| *sbmA* | 1.67 | -2.23 | 1.55 | 1.14 |
| *rpsS* | -3.46 | -11.63 | 1.30 | 1.00 |
| *rplD* | -2.54 | -11.94 | 1.71 | 1.14 |
| *rpsF* | -2.49 | -9.87 | 1.91 | -1.12 |
| *rplP* | -2.37 | -7.68 | 1.37 | 1.09 |
| *rplA* | -2.36 | -5.95 | 1.53 | -1.19 |
| *rplY* | -1.81 | -4.32 | 1.57 | -1.33 |
