# Supplementary material for: A Molecular Genetic Basis Explaining Altered Bacterial Behavior in Space
Source: PLoS One. 2016 Nov 2;11(11):e0164359. doi: 10.1371/journal.pone.0164359 (PMC5091764; doi:10.1371/journal.pone.0164359)
Supplement: S10 Table — RNA sequencing statistical data. The cut off for adjusted p-value was 0.05 with FDR correction of 0.05. (DOCX) [file pone.0164359.s010.docx]

**S10 Table. Sequencing Matrix.** RNA sequencing statistical data. The cut off for adjusted p-value was 0.05 with FDR correction of 0.05.

| **Library ID** | **Library Name** | **Total Reads** | **PF Reads** | **PF Megabases** | **% >-= Q30** | **QScore** |
| --- | --- | --- | --- | --- | --- | --- |
| SL83337 | 2904-SL-0001 | 30037664 | 17560019 | 1756 | 61.17% | 28.46 |
| SL83338 | 2904-SL-0002 | 30653274 | 20654177 | 2065 | 69.52% | 29.63 |
| SL83339 | 2904-SL-0003 | 11564204 | 7192935 | 719 | 59.83% | 28.57 |
| SL83340 | 2904-SL-0004 | 18151310 | 10678416 | 1068 | 59.92% | 28.45 |
| SL83341 | 2904-SL-0005 | 42829790 | 28918675 | 2892 | 69.86% | 29.73 |
| SL83342 | 2904-SL-0006 | 17957952 | 10523360 | 1052 | 56.40% | 27.91 |
| SL83343 | 2904-SL-0007 | 16120990 | 8990677 | 899 | 56.19% | 27.78 |
| SL83345 | 2904-SL-0009 | 57353240 | 38495495 | 3850 | 72.07% | 30.14 |
| SL83347 | 2904-SL-0011 | 17718854 | 9966856 | 997 | 56.82% | 27.97 |
| SL83348 | 2904-SL-0012 | 17447468 | 10150937 | 1015 | 56.53% | 27.97 |
| SL83393 | 2904-SL-0057 | 26183154 | 22088109 | 2209 | 75.86% | 30.77 |
| SL83394 | 2904-SL-0058 | 27525272 | 22262440 | 2226 | 75.13% | 30.63 |
| SL83395 | 2904-SL-0059 | 30976656 | 25561937 | 2556 | 75.10% | 30.56 |
| SL83396 | 2904-SL-0060 | 34666542 | 28800964 | 2880 | 75.30% | 30.61 |
| SL83397 | 2904-SL-0061 | 40310674 | 32216291 | 3222 | 73.69% | 29.95 |
| SL83399 | 2904-SL-0063 | 27415672 | 21918830 | 2192 | 72.66% | 29.81 |
| SL83400 | 2904-SL-0064 | 31633478 | 25294130 | 2529 | 73.62% | 29.87 |
| SL83401 | 2904-SL-0065 | 25040310 | 20510518 | 2051 | 74.09% | 30.19 |
| SL83402 | 2904-SL-0066 | 32848144 | 26925624 | 2693 | 73.22% | 29.75 |
| SL83403 | 2904-SL-0067 | 32601272 | 27059056 | 2706 | 74.48% | 30.1 |
| SL83404 | 2904-SL-0068 | 44455936 | 37049578 | 3705 | 74.02% | 29.61 |
