# Supplementary material for: A Molecular Genetic Basis Explaining Altered Bacterial Behavior in Space
Source: PLoS One. 2016 Nov 2;11(11):e0164359. doi: 10.1371/journal.pone.0164359 (PMC5091764; doi:10.1371/journal.pone.0164359)
Supplement: S11 Table — Gene set analysis was performed using the PANTHER over representation test (release 2016-07-15) for the candidate genes in Table 1. The candidate genes were analyzed using the Gene Ontology Database (release 2016-08-22) (GORGP, 2015). (DOCX) [file pone.0164359.s011.docx]

**S11 Table. Gene set analysis on differentially expressed genesfrom Table 1.** Gene set analysis was performed using the PANTHER over representation test (release 2016-07-15) for the candidate genes in Table 1. The candidate genes were analyzed using the Gene Ontology Database (release 2016-08-22) (GORGP, 2015).

| **GO biological process complete** | **Fold Enrichment** | **p-value** |
| --- | --- | --- |
| thiamine diphosphate metabolic process (GO:0042357) | > 100 | 2.21E-07 |
| thiamine diphosphate biosynthetic process (GO:0009229) | > 100 | 2.21E-07 |
| thiamine-containing compound biosynthetic process (GO:0042724) | > 100 | 3.41E-07 |
| thiamine biosynthetic process (GO:0009228) | > 100 | 3.41E-07 |
| thiamine metabolic process (GO:0006772) | > 100 | 1.04E-06 |
| thiamine-containing compound metabolic process (GO:0042723) | > 100 | 1.04E-06 |
| sulfur compound biosynthetic process (GO:0044272) | 47.57 | 4.44E-05 |
| pyrimidine-containing compound biosynthetic process (GO:0072528) | 38.05 | 1.34E-04 |
| pyrimidine-containing compound metabolic process (GO:0072527) | 23.78 | 1.34E-03 |
| vitamin biosynthetic process (GO:0009110) | 23.78 | 1.34E-03 |
| water-soluble vitamin biosynthetic process (GO:0042364) | 23.78 | 1.34E-03 |
| water-soluble vitamin metabolic process (GO:0006767) | 20.3 | 2.91E-03 |
| vitamin metabolic process (GO:0006766) | 20.3 | 2.91E-03 |
| sulfur compound metabolic process (GO:0006790) | 16.91 | 7.05E-03 |
| coenzyme biosynthetic process (GO:0009108) | 15.07 | 1.23E-02 |
