# Supplementary material for: A Molecular Genetic Basis Explaining Altered Bacterial Behavior in Space
Source: PLoS One. 2016 Nov 2;11(11):e0164359. doi: 10.1371/journal.pone.0164359 (PMC5091764; doi:10.1371/journal.pone.0164359)
Supplement: S12 Table — Gene set analysis was performed using the PANTHER over representation test (release 2016-07-15) for the candidate genes in Table 2. The candidate genes were analyzed using the Gene Ontology Database (release 2016-08-22) (GORGP, 2015). (DOCX) [file pone.0164359.s012.docx]

**S12 Table. Gene set analysis on differentially expressed genes from Table 2.** Gene set analysis was performed using the PANTHER over representation test (release 2016-07-15) for the candidate genes in Table 2. The candidate genes were analyzed using the Gene Ontology Database (release 2016-08-22) (GORGP, 2015).

| **GO biological process complete** | **Fold Enrichment** | **p-value** |
| --- | --- | --- |
| acetyl-CoA biosynthetic process (GO:0006085) | > 100 | 2.92E-03 |
| acetate metabolic process (GO:0006083) | > 100 | 2.92E-03 |
| fermentation (GO:0006113) | 73.48 | 2.20E-07 |
| tricarboxylic acid cycle (GO:0006099) | 65.32 | 1.93E-16 |
| thioester biosynthetic process (GO:0035384) | 62.99 | 1.54E-02 |
| acyl-CoA biosynthetic process (GO:0071616) | 62.99 | 1.54E-02 |
| acetyl-CoA metabolic process (GO:0006084) | 55.11 | 2.29E-02 |
| ADP metabolic process (GO:0046031) | 38.1 | 5.57E-07 |
| ribonucleoside diphosphate metabolic process (GO:0009185) | 36.74 | 7.15E-07 |
| purine ribonucleoside diphosphate metabolic process (GO:0009179) | 36.74 | 7.15E-07 |
| purine nucleoside diphosphate metabolic process (GO:0009135) | 36.74 | 7.15E-07 |
| glycolytic process (GO:0006096) | 36.74 | 1.33E-05 |
| aerobic respiration (GO:0009060) | 35.27 | 2.88E-13 |
| ATP generation from ADP (GO:0006757) | 35.27 | 1.70E-05 |
| nucleoside diphosphate phosphorylation (GO:0006165) | 33.92 | 2.14E-05 |
| nucleoside diphosphate metabolic process (GO:0009132) | 31.17 | 2.21E-06 |
| nucleotide phosphorylation (GO:0046939) | 28.44 | 6.00E-05 |
| pyruvate metabolic process (GO:0006090) | 27.07 | 5.79E-06 |
| ATP metabolic process (GO:0046034) | 21.43 | 2.84E-05 |
| generation of precursor metabolites and energy (GO:0006091) | 20.04 | 1.66E-21 |
| cellular respiration (GO:0045333) | 19.86 | 9.29E-14 |
| nicotinamide nucleotide metabolic process (GO:0046496) | 19.78 | 4.88E-05 |
| energy derivation by oxidation of organic compounds (GO:0015980) | 19.12 | 1.08E-14 |
| pyridine nucleotide metabolic process (GO:0019362) | 19.05 | 6.30E-05 |
| purine ribonucleoside triphosphate metabolic process (GO:0009205) | 18.37 | 8.06E-05 |
| purine nucleoside triphosphate metabolic process (GO:0009144) | 18.05 | 9.08E-05 |
| ribonucleoside triphosphate metabolic process (GO:0009199) | 17.15 | 1.28E-04 |
| pyridine-containing compound metabolic process (GO:0072524) | 16.86 | 1.43E-04 |
| nucleoside triphosphate metabolic process (GO:0009141) | 15.13 | 2.97E-04 |
| oxidoreduction coenzyme metabolic process (GO:0006733) | 15.13 | 2.97E-04 |
| purine ribonucleoside monophosphate metabolic process (GO:0009167) | 14.49 | 3.96E-04 |
| purine nucleoside monophosphate metabolic process (GO:0009126) | 14.49 | 3.96E-04 |
| protein complex assembly (GO:0006461) | 12.25 | 4.87E-02 |
| protein complex biogenesis (GO:0070271) | 12.25 | 4.87E-02 |
| purine ribonucleoside metabolic process (GO:0046128) | 11.96 | 1.42E-03 |
| ribonucleoside monophosphate metabolic process (GO:0009161) | 11.82 | 1.53E-03 |
| purine ribonucleotide metabolic process (GO:0009150) | 11.69 | 1.65E-03 |
| purine nucleoside metabolic process (GO:0042278) | 11.31 | 2.05E-03 |
| nucleoside monophosphate metabolic process (GO:0009123) | 11.18 | 2.21E-03 |
| dicarboxylic acid metabolic process (GO:0043648) | 11.16 | 1.31E-02 |
| purine nucleotide metabolic process (GO:0006163) | 11.06 | 2.37E-03 |
| ribonucleotide metabolic process (GO:0009259) | 9.61 | 5.93E-03 |
| ribonucleoside metabolic process (GO:0009119) | 8.95 | 9.48E-03 |
| purine-containing compound metabolic process (GO:0072521) | 8.57 | 1.25E-02 |
| nucleoside metabolic process (GO:0009116) | 8.5 | 1.32E-02 |
| coenzyme metabolic process (GO:0006732) | 8.48 | 5.96E-04 |
| glycosyl compound metabolic process (GO:1901657) | 8.36 | 1.46E-02 |
| ribose phosphate metabolic process (GO:0019693) | 8.36 | 1.46E-02 |
| cellular component assembly (GO:0022607) | 7.79 | 5.37E-03 |
| monocarboxylic acid metabolic process (GO:0032787) | 7.74 | 5.88E-05 |
| organic acid metabolic process (GO:0006082) | 6.53 | 1.98E-13 |
| cofactor metabolic process (GO:0051186) | 6.07 | 9.25E-03 |
| oxidation-reduction process (GO:0055114) | 5.84 | 9.27E-10 |
| single-organism carbohydrate metabolic process (GO:0044723) | 5.25 | 2.80E-03 |
| carboxylic acid metabolic process (GO:0019752) | 5.09 | 6.01E-06 |
| oxoacid metabolic process (GO:0043436) | 4.85 | 1.22E-05 |
| small molecule metabolic process (GO:0044281) | 4.3 | 3.59E-11 |
| carbohydrate metabolic process (GO:0005975) | 4.28 | 1.99E-02 |
| single-organism metabolic process (GO:0044710) | 2.95 | 5.57E-09 |
| single-organism cellular process (GO:0044763) | 2.44 | 2.26E-05 |
| single-organism process (GO:0044699) | 2.01 | 1.98E-05 |
| cellular metabolic process (GO:0044237) | 1.84 | 4.36E-03 |
| organic substance metabolic process (GO:0071704) | 1.76 | 3.33E-02 |
| metabolic process (GO:0008152) | 1.6 | 8.87E-03 |
