# Supplementary material for: A Molecular Genetic Basis Explaining Altered Bacterial Behavior in Space
Source: PLoS One. 2016 Nov 2;11(11):e0164359. doi: 10.1371/journal.pone.0164359 (PMC5091764; doi:10.1371/journal.pone.0164359)
Supplement: S13 Table — Gene set analysis was performed using the PANTHER over representation test (release 2016-07-15) for the candidate genes in Table 3. The candidate genes were analyzed using the Gene Ontology Database (release 2016-08-22) (GORGP, 2015). (DOCX) [file pone.0164359.s013.docx]

**S13 Table. Gene set analysis son differentially expressed genes from Table 3.** Gene set analysis was performed using the PANTHER over representation test (release 2016-07-15) for the candidate genes in Table 3. The candidate genes were analyzed using the Gene Ontology Database (release 2016-08-22) (GORGP, 2015).

| **GO biological process complete** | **Fold Enrichment** | **p-value** |
| --- | --- | --- |
| pH elevation (GO:0045852) | 73.06 | 1.01E-02 |
| intracellular pH elevation (GO:0051454) | 73.06 | 1.01E-02 |
| regulation of intracellular pH (GO:0051453) | 60.89 | 1.74E-02 |
| regulation of cellular pH (GO:0030641) | 45.66 | 4.07E-02 |
| response to pH (GO:0009268) | 27.06 | 8.82E-05 |
| response to abiotic stimulus (GO:0009628) | 6.28 | 2.93E-02 |
| response to stimulus (GO:0050896) | 2.83 | 3.81E-03 |
